# Two auxins are better than one: BiAux joins forces with auxin to enhance lateral root formation

**DOI:** 10.1093/plphys/kiae132

**Published:** 2024-03-06

**Authors:** Héctor H Torres-Martínez

**Affiliations:** Assistant Features Editor, Plant Physiology, American Society of Plant Biologists; Department of Biology, Stanford University, Stanford, CA 94305, USA

The iterative formation of lateral roots (LRs) during plant life span allows the exploration of new sources of nutrients and water. Auxin plays a key role in all major stages of LR formation. Auxin signal is required for priming pericycle cells into LR founder cells (FC), LR primordium initiation, morphogenesis, and emergence (Du and Scheres 2018). In *Arabidopsis thaliana*, LRs are set along the primary root by a mechanism known as “root clock,” where a periodic gene expression in phase and antiphase with an auxin signal peak oscillates in a position close to the primary root tip, named the oscillation zone (OZ). This oscillatory behavior results in the establishment of a pre-branch site, a position where pericycle cells with a memorized priming signal initiate an LR primordium later on (Reyes-Hernández and Maizel 2023).

During LR development, auxin signal is mainly transduced through the formation of a co-receptor by TRANSPORT INHIBITOR RESPONSE1 (TIR1) or its closest paralogues, AUXIN SIGNALING F-BOX (AFB1 to AFB5) proteins and Aux/IAA family proteins, where auxin plays a role as the molecular glue for this assembling (Tan et al. 2007). When low levels of auxin are present in cells, Aux/IAA proteins tend to dimerize with AUXIN RESPONSE FACTOR (ARF) transcription factor family members, repressing ARF activity. Once auxin levels are increased, Aux/IAA proteins are targeted for degradation, releasing ARF proteins to regulate the transcription of their target genes (Zenser et al. 2001; Tiwari et al. 2003).

A number of Aux/IAA-ARF modules are known to participate during LR development (Du and Scheres 2018). Arabidopsis has 6 TIR1/AFBs (Gagne et al. 2002), 29 Aux/IAA proteins, and 23 ARF members (Remington et al. 2004). Different combinations of ARF, Aux/IAA, and AFB can result in a myriad of auxin response modules associated with specific plant developmental processes. Understanding the precise mechanism of how co-receptors or Aux/IAA modules are assembled will shed light on the mechanisms by which auxin regulates development *in planta*.

In this issue of *Plant Physiology*, González-García et al. (2024) identified a novel compound, BiAux, which is accumulated in roots exposed to light. BiAux binds to a specific site in TIR1/AFBs proteins and, together with auxin, enhances the sensitivity to auxin signaling, increasing pre-branch sites along the primary root (Figure). Using markers for both cell division, p*SKP2B::GUS* (Manzano et al. 2012), and auxin response, p*DR5::LUC* (Ulmasov et al. 1997), authors found that BiAux-treated seedlings increased the number of LR initiation events in a dose- and exposure time–dependent manner. Time-course experiments in p*DR5::LUC* seedlings showed a BiAux-mediated increased auxin response signal amplitude in the OZ that correlated with more and closer pre-branch sites (Figure). In addition, established pre-branch sites kept a sustained auxin response signal in 100% of the cases.

**Figure.**
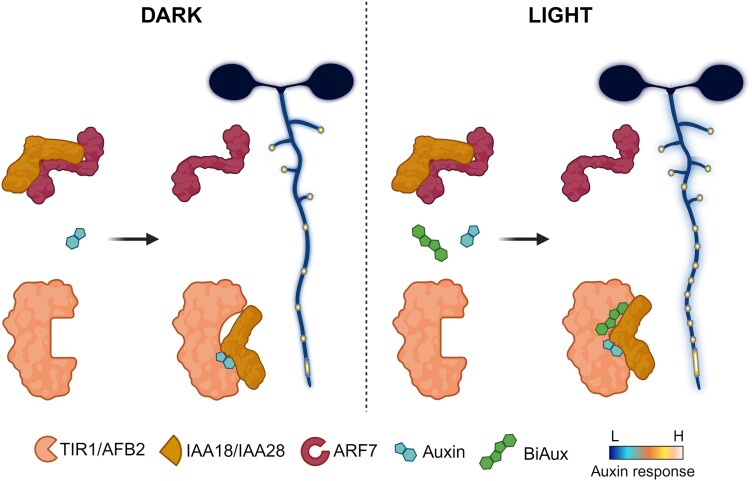
A proposed model for BiAux action mechanism in LR formation. The traditional model for auxin signal transduction explains that auxin works as a molecular glue that promotes co-receptor assembly between TIR/AFB and Aux/IAA protein family members. In this new research piece, González-García et al. (2024) described the isolation and characterization of a new metabolite, BiAux, which is accumulated when roots are grown under illumination, such as on traditional Petri dishes. It was demonstrated that BiAux binds to a specific TIR1 site close to the auxin binding site, and the binding seems to stabilize the co-receptor complex. Specifically, simultaneous treatment with auxin and BiAux enhanced the co-receptor formation between TIR1 or AFB2 and IAA18 or IAA28, auxin signal regulators for early LR formation. These complexes might release the positive auxin signal regulator ARF7, which was found to mediate the BiAux response during the lateral formation process. Figure was created with BioRender.com by H.H.T.M.

Interestingly, different auxin signaling mutants responded to BiAux rather differently. For example, BiAux increased the density of the LR in *auxin-resistan1* (*axr1-12*) mutant, which is affected in the activation of Skp, Cullin, and F-box containing complex (or SCF complex) of TIR1. In *solitary root1* (*slr1*) mutant, which harbors a dominant mutation in the *IAA14* gene, BiAux only increased the number of FC spots. Moreover, while BiAux treatment resulted in a higher number of LR initiation events in *tir1/afb* single mutants, there a significant reduction of LR initiation events only in the *tir1*/*afb2* double mutant or in higher-order mutants that harbored the *tir1* and *afb2* alleles. Together, this indicated the additive effect of BiAux in auxin signaling and the pivotal role of TIR1 and AFB2 in mediating the BiAux response. In agreement, transcriptomics analysis using BiAux, auxin, or a combined treatment showed that BiAux altered the expression level of genes related to root development and hormone signaling regulated by auxin. Additionally, docking analysis indicated that BiAux binds to a pocket near the auxin binding site in the TIR1 protein. Due to the importance of these residues for TIR1 and Aux/IAA proteins interaction, BiAux is suggested to stabilize the co-receptor formation (Figure).

To further study the action mechanisms of BiAux, the authors analyzed available transcriptomics databases and found that the co-regulated genes by BiAux and auxin during LR initiation were not induced or induced at a lesser extent in the *auxin response factor7* (*arf7*) mutant compared to wild type. BiAux treatment in *arf7* did not increase the number of LR initiation events, suggesting that ARF7 is required for BiAux-mediated LR development (Figure).

Furthermore, the transcriptomic dataset also showed that BiAux mainly deregulates overlapped genes with auxin response, specifically with those in phase-upregulated genes, suggesting its role in the root clock. Analysis of BiAux treatment in *potent*, a dominant mutant of *IAA18/POTENT* and a negative regulator of the root clock, showed an increased auxin response signal amplitude in the OZ. On the other hand, when *iaa28*, a dominant mutant impaired in FC specification, was treated with BiAux it increased the number of initiation events, although to a lesser extent compared to wild type. Yeast 2-hybrid interaction essays demonstrated that in the presence of auxin, BiAux enhances the interaction of auxin co-receptor only between IAA18/POTENT and TIR1, AFB1 or AFB2, whereas IAA28 interaction is only enhanced with TIR1 or AFB2. However, BiAux alone did not stabilize the interaction between TIR1/AFBs and IAAs. CLSM time-lapse experiments in seedlings expressing an IAA28-VENUS fusion confirmed that BiAux, in combination with auxin, promotes the IAA28 degradation. Together, these findings show that BiAux favors the formation of specific auxin co-receptors during LR formation (Figure).

Despite the vast efforts made to understand the LR developmental program in the model plant *A. thaliana*, González-García et al. (2024) remind us of many unsolved questions. The new findings reported the role of BiAux, a light-induced metabolite that helps stabilize specific auxin signaling co-receptors together with auxin, thus enhancing the LR formation (Figure). Nonetheless, this valuable research piece raises exciting questions that remain unanswered, such as how this compound is synthesized and transported in planta and in what other auxin-dependent mechanisms it is involved. Undoubtedly, BiAux will help to gain more insights into the complex “root clock” biology and how the environment may tune it.

## Dive Curated Terms

The following phenotypic, genotypic, and functional terms are of significance to the work described in this paper:

AFB5 Gramene: AT5G49980
AFB5 Araport: AT5G49980

IAA14 Gramene: AT4G14550
IAA14 Araport: AT4G14550

AFB2 Gramene: AT3G26810
AFB2 Araport: AT3G26810

IAA28 Gramene: AT5G25890
IAA28 Araport: AT5G25890

AFB1 Gramene: AT4G03190
AFB1 Araport: AT4G03190

TIR1 Gramene: AT3G62980
TIR1 Araport: AT3G62980

IAA18 Gramene: AT1G51950
IAA18 Araport: AT1G51950

axr1-12 Gramene: AT1G05180
axr1-12 Araport: AT1G05180
